# Bibliometric Differences Between WEIRD and Non-WEIRD Countries in the Outcome Research on Solution-Focused Brief Therapy

**DOI:** 10.3389/fpsyg.2021.754885

**Published:** 2021-11-17

**Authors:** Mark Beyebach, Marie-Carmen Neipp, Ángel Solanes-Puchol, Beatriz Martín-del-Río

**Affiliations:** ^1^Department of Health Sciences, Public University of Navarre, Pamplona, Spain; ^2^Department of Health Psychology, University of Miguel Hernández, Elche, Spain; ^3^Department of Behavioral Sciences and Health, University of Miguel Hernández, Elche, Spain

**Keywords:** solution-focused brief therapy, solution-focused therapy, positive psychology, WEIRD, non-WEIRD, bibliometric analysis

## Abstract

Solution Focused Brief Therapy (SFBT) developed in parallel to Positive Psychology, as a type of intervention that also emphasizes the strengths and resources of clients. The aim of this study was to examine the development of outcome research on SFBT and to determine whether it is predominantly carried out in Western, Educated, Industrialized, Rich and Democratic (WEIRD) countries. A literature review was conducted using a bibliometric methodology, identifying: (a) authors and countries, (b) time trends, (c) language of publications; (d) and journals; (e) samples on which they were tested; (f) characteristics of interventions; and (g) main study designs. A total of 365 original outcome research articles published in scientific journals on solution-focused interventions were extracted. The results show that outcome research on SFBT has grown steadily over the last three decades. Although it started in WEIRD countries, the number of outcome research publications generated in non-WEIRD countries is now higher. There is little international collaboration and, although English is the main language of publication in WEIRD countries, English, Chinese and Parsi predominate in non-WEIRD countries. Productivity is low and most authors have only published one paper. The journals that have published the most papers have a very diverse visibility. The tested interventions are conducted both in clinical and non-clinical samples; mostly in individual and group format; face-to-face; and not only in the form of psychotherapy, but also as coaching and school interventions. Almost half of the publications are randomized controlled trials. The results confirm the wide applicability of SFBT as a single or main component of psychosocial interventions. They support the claim that solution-focused interventions are not a WEIRD practice, but a global practice.

## Introduction

Solution-focused Brief Therapy (SFBT) is a therapeutic approach that developed outside the Positive Psychology field but shows several fundamental coincidences with it. SFBT was created by Steve de Shazer, Insoo Kim Berg and a group of enthusiastic social workers in Milwaukee, Wisconsin in the eighties, years before the official creation of the Positive Psychology field (Seligman, 1999). SFBT developed within the strategic tradition of brief family therapy (Weakland et al., 1982), initially as a way to complement its narrow focus on interactional problem patterns (de Shazer et al., 1986), but evolved into a radical approach that changed the therapy focus from problems to what was called “solutions”: exceptions to the problems, strengths, improvements and goals (de Shazer, 1994; de Shazer et al., 2007). The emphasis on the strengths and resources of clients, and the straightforward nature of the approach, lead to its expansion to a number of intervention contexts beyond psychotherapy and family therapy: social work (Sundman, 1997), child protection (Berg and Kelly, 2000), coaching (Berg and Szabó, 2005), nursing (MCAllister, 2007), organizational consulting (McKergow, 2012), mediation (Bannink, 2007), pastoral work (Kollar, 1997), school counseling (Kelly et al., 2008), or University teaching (Devlin, 2003), among others. Over the last decades, SFBT has amassed considerable evidence of its effectiveness and cost-efficiency in a variety of contexts (Kim, 2008, 2012; Bond et al., 2013; Gingerich and Peterson, 2013; Kim et al., 2015, 2019; Carr et al., 2016; Gong and Hsu, 2017), demonstrating outcomes equivalent to those of alternative interventions, both at termination (e.g., Creswell et al., 2017) and at follow-up (e.g., Boyer et al., 2015).

The similarities and complementarities between Positive Psychology (PP) and the solution-focused approach have been pointed out by a number of scholars (Glass, 2009; Bannink and Jackson, 2011; Warner, 2013; Blundo et al., 2014; Pereira et al., 2017). At the level of practice, solution-focused therapists co-construct solutions in dialogue with their clients by focusing on their desired futures and those occasions when parts of those futures are already happening, capitalizing on clients’ strengths and past successes instead of analyzing problems and their causes. SFBT is therefore not a problem-solving procedure, but a process of solution construction. At a conceptual level, the solution-focused approach shares with Positive Psychology the trust in the capabilities and strengths of people, the rejection of the “illness ideology” (Maddux, 2009) and the deconstruction of diagnostic labels (de Shazer and Berg, 1991). SFBT is not construed as specific therapies for specific “disorders,” but as a general procedure that can help all kind of clients achieve their own goals. The solution-focused emphasis on collaboration with clients and on “leading from one step behind” (Cantwell and Holmes, 1994) is consistent with the promotion of clients’ self-determination (Deci and Ryan, 2002) and self-efficacy (Maddux, 2009); the position of curiosity and humility that solution-focused practitioners adopt vis a vis their clients resonates with Positive Psychology’s emphasis on the character strengths and virtues of people (Peterson and Seligman, 2004). The recent emphasis in the SFBT literature on the emotional side of SFBT interventions, and specifically on the role of positive emotions in promoting therapeutic in-session change (Connie, 2013; Kim and Franklin, 2015; Neipp et al., 2016) is another parallel with Positive Psychology interests (Fredrickson, 2001).

There are also some important differences among PPIs (Positive Psychology Interventions) and SFBT interventions. The most salient one is that in the solution-focused approach there is no aspiration to propose a universal model of psychological wellbeing or to promote a given recipe for happiness or growth, as is the case in PP (for instance, PERMA, Seligman, 2018). Instead, SFBT takes a constructivist and non-expert approach to wellbeing that translates into a constant effort to adjust to the individual person, to respect their worldviews and use their values and believes as resources for change. In our view, this makes SFBT especially suited to work within different cultural contexts and to intervene with cultural minorities and specific communities (Kim, 2013; Ouer, 2016). Another difference is that SFBT comes from a “hands on,” action-oriented social work tradition, outside the world of academia and university-based research in which PP is rooted. Furthermore, SFBT developed as a brief intervention to construct workable, as simple as possible solutions in difficult contexts. In our view, the solution-focused emphasis on simplicity and the use brief interventions also increases its applicability with under-privileged populations. Another difference is that SFBT is far more homogeneous than PPIs. While PPIs include a number of very different practices, from positive recollections and positive psycho education to gratitude expression, mindfulness or life review (Hendriks et al., 2018), all SFBT interventions include, in one way or another, the same basic elements of the solution-building process.

These differences between PPIs and SFBT may give SFBT an advantage in terms of how applicable it is worldwide, beyond the limits of western countries. Different authors indicate that PPIs are too Western-centric (Christopher and Hickinbottom, 2008; Frawley, 2015) since the origin of Positive Psychology is linked to the North American culture. In PP, happiness and flourishing are constructed as an individual process, assuming social and cultural values of that region, underestimating the importance of social, cultural and historical factors of other countries. This is evidenced in a recent systematic review by Kim et al. (2018) who conclude that 78% of the research in Positive Psychology has been conducted in Western countries. Moreover, the bibliometric study carried out by Hendriks et al. (2018) reflects that 78.2% of Randomized Controlled Trials (RCTs) on the efficacy of PPI have been conducted in WEIRD countries. Most of the samples represented in these studies are WEIRD samples (Western, Educated, Industrialized, Rich and Democratic; Henrich et al., 2010a,b) and do not represent the characteristics of the majority of the world’s population. However, since 2012 there has been a strong increase in publications on PPI from non-Western countries, indicating a promising trend of expansion of positive psychology research globally (Hendriks et al., 2018).

This study examined the differences between WEIRD and non-WEIRD countries in the worldwide scientific production on SFBT. To this end, a bibliometric study of the literature on SFBT outcome research was carried out, in which (a) authors and countries, (b) time trends, (c) language of the publications; (d) and journals were identified; (e) the samples on which SFBT were tested; (f) the features of the SFBT interventions (use of SFBT, format of implementation, type of intervention and modality of intervention); and (g) the main study designs of research on this type of interventions were also analyzed. Based on the differences between PP and SFBT, we expected to find a more balanced WEIRD/non-WEIRD production on SFBT interventions than on PPIs.

## Methods

### Search Methods

A systematic literature search was conducted by BMR and ASP from May 29th to May 31st, 2021, in nine databases: Web of Science Core Collection (WOSCc), Medline, Scopus, PsycINFO, ERIC, Embase, PubMed, ASSIA y SciELO. The databases were searched with the following terms, adapted to each database: solution focused brief therapy OR solution focused therapy. The search was done on the titles, abstracts and keywords of articles, without any restrictions on dates, language or availability. In addition, all articles included in the data base on research on Solution Focused Brief Therapy of the Solution Focused Brief Therapy Association (SFBTA)^1^ were reviewed.

### Search Outcomes and Eligibility Criteria

2,251 records were initially identified. After removal of duplicates, 1,144 remained. MCN performed a first reading of the titles and abstracts, eliminating another 528 records. Afterward, MCN and MB reviewed the whole data base, selecting 365 records for the bibliometric analysis. The few disagreements between the two authors were discussed and solved by consensus.

**FIGURE 1:**
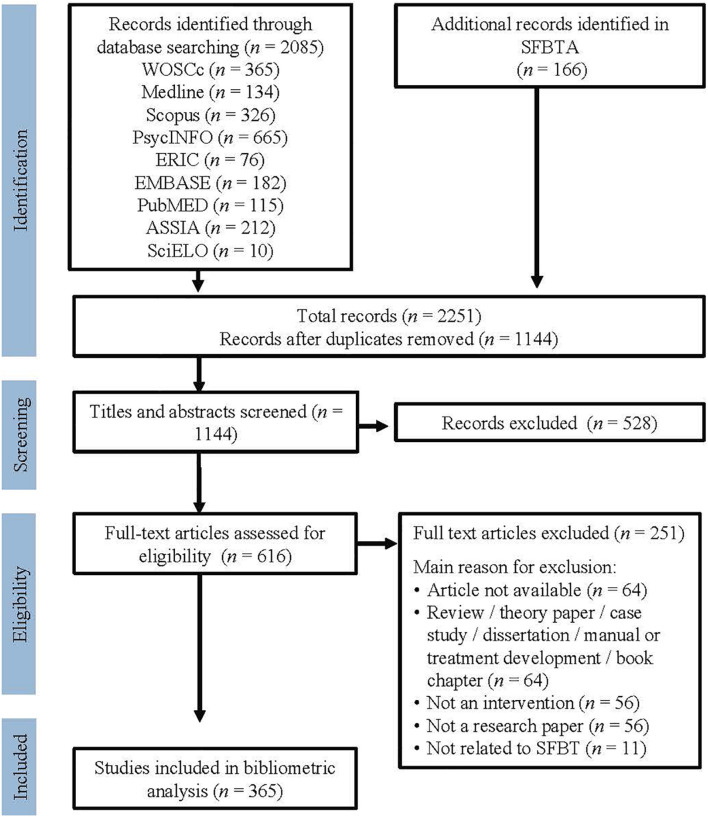
Flow chat through the different phases of the literature review.

The same inclusion and exclusion criteria were used at all stages of the selection process. Inclusion criteria were: (a) original research articles, (b) published in scientific journals, (c) on the outcome (effectiveness or efficacy) of psychosocial interventions in which (d) at least one component was solution-focused. We excluded: (a) non-original research papers, (b) research papers that did not focus on interventions, and (c) research papers that focused only on the process of a SFI (not on its outcome). Papers with non-accessible content were also excluded (Figure 1).

### Data Extraction and Analysis

Once all original papers had been retrieved, BMR, ASP and MCN extracted their titles, abstracts, authors, affiliations, publication year and journal. Information on the design of the study, the sample (type of participants and sample size) and the intervention (type, context, and format) was extracted by MCN and MB.

MCN and BMR classified the articles as proceeding from WEIRD or non-WEIRD countries according to the affiliation of their first author. Hendriks et al.’s (2018) criteria were used: (1) Western: countries in North America, Western Europe, Israel, Australia and New Zealand were classified as Western societies. (2) Educated: human development was rated as very high, high, average, or low according to the United Nations Development Programme (2020). (3) Industrialized: countries were classified as advanced or emerging economies, according to the International Monetary Fund (2021). (4) Rich: The Global Wealth Databook (Credit Suisse, 2020) was the basis to classify countries as high, upper middle, lower middle and low income. (5) Democratic: countries were classified as a full democracy, a flawed democracy, a hybrid regime or an authoritarian regime according to the Democracy Index compiled by aaa The Economist Intelligence Unit [EIU], 2007, 2008, 2010, 2011, 2012, 2013, 2014, 2015, 2016, 2017, 2018, 2019, 2020. The Democracy Index is subject to temporal variations due to conjunctural political changes. For this reason, we used the average Democracy Index score from 2006 to 2020. Countries were classified as WEIRD if they met all five (see Table 1).

**TABLE 1:** Description of WEIRD parameters by country and classification.

**Country**	**W^1^**	**E^2^**	**I^3^**	**R^4^**	**D^5^**	**Classification**
Australia	Western	Very high	Advanced	High	Full	WEIRD
Bolivia	Non-Western	High	Emerging	Lower middle	Hybrid	Non-WEIRD
Canada	Western	Very high	Advanced	High	Full	WEIRD
Chile	Non-Western	Very high	Emerging	Upper middle	Full	Non-WEIRD
China	Non-Western	High	Emerging	Upper middle	Authoritarian	Non-WEIRD
Colombia	Non-Western	High	Emerging	Lower middle	Flawed	Non-WEIRD
Finland	Western	Very high	Advanced	High	Full	WEIRD
Germany	Western	Very high	Advanced	High	Full	WEIRD
Greece	Western	Very high	Advanced	High	Flawed	Non-WEIRD
India	Non-Western	Medium	Emerging	Lower middle	Flawed	Non-WEIRD
Indonesia	Non-Western	High	Emerging	Lower middle	Flawed	Non-WEIRD
Iran	Non-Western	High	Emerging	Lower middle	Authoritarian	Non-WEIRD
Ireland	Western	Very high	Advanced	High	Full	WEIRD
Japan	Non-Western	Very high	Advanced	High	Full	Non-WEIRD
Jordan	Non-Western	High	Emerging	Upper middle	Authoritarian	Non-WEIRD
Lithuania	Eastern Europe	Very high	Advanced	High	Flawed	Non-WEIRD
Mexico	Non-Western	High	Emerging	Upper middle	Flawed	Non-WEIRD
Netherlands	Western	Very high	Advanced	High	Full	WEIRD
New Zealand	Western	Very high	Advanced	High	Full	WEIRD
Nigeria	Non-Western	Low	Emerging	Low	Hybrid	Non-WEIRD
Norway	Western	Very high	Advanced	High	Full	WEIRD
Peru	Non-Western	High	Emerging	Lower middle	Flawed	Non-WEIRD
Poland	Eastern Europe	Very high	Advanced	High	Flawed	Non-WEIRD
Romania	Eastern Europe	Very high	Advanced	High	Flawed	Non-WEIRD
South Africa	Non-Western	Medium	Emerging	Lower middle	Flawed	Non-WEIRD
South Korea	Non-Western	Very high	Advanced	High	Full	Non-WEIRD
Spain	Western	Very high	Advanced	High	Full	WEIRD
Sweden	Western	Very high	Advanced	High	Full	WEIRD
Taiwan	Non-Western	Very high	Advanced	High	Flawed	Non-WEIRD
Thailand	Non-Western	High	Emerging	Upper middle	Flawed	Non-WEIRD
Turkey	Non-Western	Very high	Emerging	Upper middle	Hybrid	Non-WEIRD
United Kingdom	Western	Very high	Advanced	High	Full	WEIRD
United States	Western	Very high	Advanced	High	Full	WEIRD

*^1^W, region.*

*^2^E, educated (human development).*

*^3^I, industrialized (economy).*

*^4^R, rich (income).*

*^5^D, democratic.*

Bradford’s Law (Bradford, 1934; Brookes, 1969) was used to classify the journals that published the retrieved papers according to three groups of decreasing productivity. Each group contains an approximate number of articles that have been published by a decreasing number of journals. This allows the determination of a first group of journals with the highest production and two others with the lowest productivity in geometric progression. Price’s transience index, [(number of authors with only one publication/total number of authors) × 100] was the used to evaluate the proportion of authors with only one publication.

All data obtained were stored and descriptively analyzed with Microsoft Excel. In addition, chi-square analysis and Student’s *t*-test were performed with the IBM SPSS Statistics 26 to compare the proportions of different sample types and of different intervention features in WEIRD and non-WEIRD countries.

## Results

### General Bibliometrics

**FIGURE 2:**
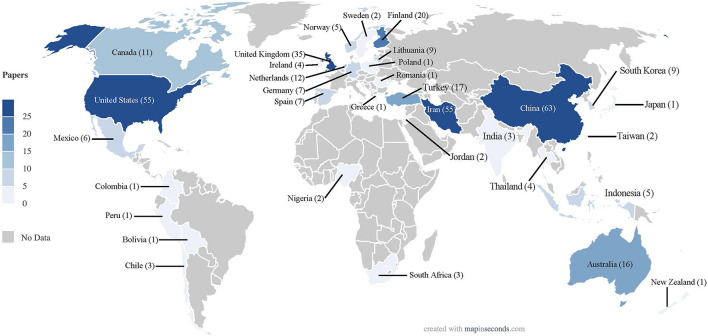
Geographical distribution of selected publications.

The 365 outcome research articles on SFBT originated from 12 WEIRD and from 21 non-WEIRD countries in all five continents. Of the 365 studies, 175 (47.95%) were conducted in WEIRD countries and 190 (52.05%), in non-WEIRD ones (Figure 2). By continents, 44.11% of the papers originated from Asia, with China, Iran, Turkey y South Korea accounting for 39.45% of the studies; twelve European countries made the second largest contribution (28.49%), originating mostly in United Kingdom, Finland, Netherlands and Lithuania (20.82%). Seven American countries accounted for 21.37% of the studies, most of them conducted in United States and Canada (18.08%); Oceania (4.66%) and Africa (1.37%) were only marginal contributors. Seventy-five percent of all the studies originated in only eight of 33 contributing countries, five of them WEIRD (United States, United Kingdom, Finland, Australia, and Netherlands) and three of them non-WEIRD (China, Iran, and Turkey) countries.

As far as international cooperation in concerned, only 17 retrieved studies were conducted by authors of different countries. Nine of these international collaborations included authors from different WEIRD countries, most of them European, and eight publications had author affiliations from WEIRD and non-WEIRD countries.

**FIGURE 3:**
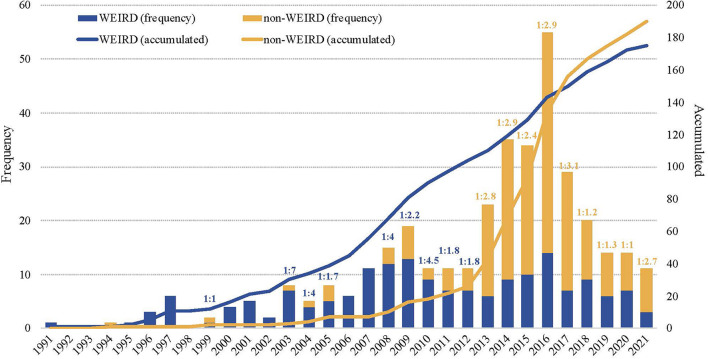
Temporal distribution of publications, accumulated frequency and WEIRD/non-WEIRD publication ratio.

Outcome research on SFBT has been published from 1991 to 2021 (*Min*. = 1; *Max.* = 55), with a slow progression up to 2006 (*n* = 52; Mean = 3.25), a constant growth until 2016 (*n* = 225; Mean = 22.5) and some decline after that (*n* = 88; Mean = 17.6) (Figure 3). The first outcome studies on SFBT were published in WEIRD countries during the nineteenths, with an irregular progression over the next 30 years. Although research on SFBT in non-WEIRD countries began in 1994 with a paper from Greece, it was not until a decade later that a real start took place. The non-WEIRD outcome research shows a more regular progression than the WEIRD production, accelerating between 2013 and 2017, when it reached the level of accumulated publications of WEIRD countries. From 2003 (when research on SFBT started to be published in non-WEIRD countries) until 2012 the average ratio of non-WEIRD vs. WEIRD publications was 1:3.4. From 2013 on, the number of yearly publications in non-WEIRD countries has doubled the production of WEIRD countries, with an average ratio of 2.3:1 (see Figure 3).

All studies conducted in WEIRD countries were published in English (93.09%). In non-WEIRD publications, the most used languages were English (35.59%), Chinese (31.64%) and Parsi (19.77%); and to a lesser extent Spanish (5.08%), Korean (5.08%), Turkish (4.52%), Indonesian (2.26), Thai (2.26%), Japanese (0.56%), and Lithuanian (0.56%).

Publications on the outcomes of SFBT were authored by 969 different contributors, with a range of 1–11 authors per publication. These authors signed 1,251 times, with an average of 3.4 authors per publication. 45.48% of authorships corresponded to non-WEIRD, and 54.52% to WEIRD countries. Only five authors were great producers, with 10 or more publications (see Table 2). A high transience rate of 84.52% was found, with 819 of the 969 authors participating in only one publication. This indicates that the majority were occasional authors in this field. Finally, of the 14 authors with five or more publications, only three were from non-WEIRD countries; the affiliations of the first nine authors in order of productivity were all in WEIRD countries.

**TABLE 2:** Most productive authors.

**Author**	**Articles**	**Institution**	**Country**
Paul B. Knekt	19	Finnish Institute for Health and Welfare	Finland
Olavi Lindfors	19	Finnish Institute for Health and Welfare	Finland
Esa Virtala	12	Finnish Institute for Health and Welfare	Finland
Cynthia G. S. Franklin	11	The University of Texas at Austin	United States
Maarit Laaksonen	10	Finnish Institute for Health and Welfare	Finland
Mark Beyebach	8	Univ. Pública Navarra and Univ. Pontificia Salamanca	Spain
Anthony M. Grant	8	University of Sydney	Australia
Tommi Härkänen	8	Finnish Institute for Health and Welfare	Finland
Erkki Heinonen	7	Finnish Institute for Health and Welfare	Finland
Viktorija Cepukiene	5	Vytautas Magnus University	Lithuania
David Alexander Grone	5	Goethe-Univeresität Frankfurt am Main	Germany
Stefanie Mache	5	Universitätsklinikum Hamburg-Eppendorf	Germany
Rytis Pakrosnis	5	Vytautas Magnus University	Lithuania
Abdollah Shafiabadi	5	Islamic Azad Univ. and Allameh Tabataba’i Univ.	Iran

Solution focused brief therapy outcome studies have been published in 261 different journals. Applying Bradford’s Law (Bradford, 1934; Brookes, 1969), 11 journals are the most productive ones, having published four or more articles on SFBT outcomes (*n* = 69; 18.90%). In these 11 journals, 72.46% of the publications come from WEIRD and 27.54% from non-WEIRD countries (see Table 3). As far as their visibility is concerned, eight of the 11 most productive journals were indexed in the WOS and SCOPUS databases, and seven in the JCR 2020 edition. However, the most productive journal, the *Chinese Journal of Modern Nursing*, is not indexed in any of these three, while the *Journal of Systemic Therapies* and *Modern Nursing* are only indexed in Google Scholar.

**TABLE 3:** Journals, papers published in WEIRD and non-WEIRD countries and presence in databases.

**Journal**	**n**	**WEIRD**	**Non- WEIRD**	**WOS**	**Scopus**	**Google Scholar**	**JCR^2^**
Chinese J. of Modern Nursing	11	0	11	No	No	No	–
J. of Systemic Therapies	9	8	1	No	No	Yes	–
Research on Social Work Practice	7	5	2	Yes	Yes	Yes	Q1
J. of Affective Disorders	6	6	0	Yes	Yes	Yes	Q2
J. of Family Psychotherapy	6	5	1	Yes	Yes	Yes	Q4
J. of Marital and Family Therapy	6	6	0	Yes	Yes	Yes	Q3
J. of Psychiatric and Mental Health Nursing	6	6	0	Yes	Yes	Yes	Q3
J. of Family Therapy	5	5	0	Yes	Yes	Yes	Q4
Modern Nursing	1	4	5	No	No	Yes	–
Children and Schools	4	0	4	Yes	Yes	Yes	–
J. of Positive Psychology	4	0	4	Yes	Yes	Yes	Q1
First area of productivity^1^	11/69	51	18				
Second area of productivity^1^	38/84	49	45				
Third area of productivity^1^	212/212	88	124				

*^1^Distribution of journals and papers according to Bradford’s area of productivity and WEIRD and non-WEIRD countries.*

*^2^Quartile of the Journal in its category in the Journal citation Reports (JCR) 2020.*

### Samples in the Retrieved Studies

In the retrieved SFBT studies, samples ranged from 1 to 3,910 subjects, with an average of 98.13 and a Median of 148.13. The average sample size was 135.27 (SD = 379.52) in WEIRD studies and 63.54 (SD = 53.72) in non-WEIRD studies, and it was significantly different (*t* = 2.610; *p* = 0.000). Taking only RCTs into account, the average sample size was 212.03 (SD = 395.59) in WEIRD studies and 73.93 (SD = 41.29) in non-WEIRD studies, also a significant difference (*t* = 3.678, *p* = 0.000).

**FIGURE 4:**
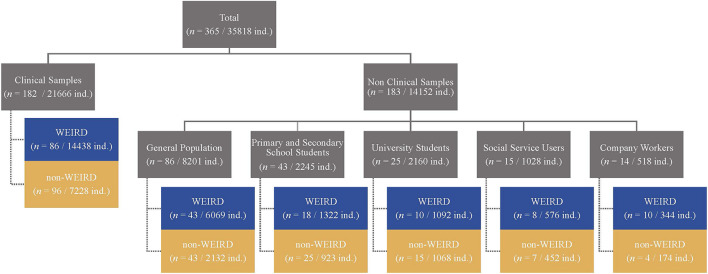
Number of papers and sample size with different types of participants in WEIRD and non-WEIRD studies.

Of the 365 papers on the outcome of SFBT, 182 studies had clinical samples (60.49% of all subjects) (see Figure 4). Of these, 86 (40.31% of subjects) were done by researchers from WEIRD countries and 96 (20.18% of subjects) were conducted by researchers from non-WEIRD countries.

The remaining 183 studies (39.51% of all subjects) were conducted on a wide range of non-clinical samples (see Figure 4): (a) General population (*n* = 86; 8,201 subjects), with the same number of papers in WEIRD and non-WEIRD countries (50% of papers and 74% of subjects from WEIRD countries); (b) primary and secondary school students (*n* = 43; 2,245 subjects), with a larger sample in the non-WEIRD country papers (58.14% of papers and 41.11% of subjects); (c) university students (*n* = 25; 2,160 subjects), with more papers published in non-WEIRD countries but similar samples (60% of papers and 49.44% of subjects); (d) social service users (*n* = 15; 1,028 subjects), with a majority of WEIRD countries (53.33% of papers and 56.03% of subjects); and (e) company workers (*n* = 14; 518 subjects), also with a majority of WEIRD countries (71.43% of papers and 66.41% of subjects). Globally, the differences between WEIRD and non-WEIRD countries in the distribution of sample types were not statistically significant.

### Features of the Solution Focused Brief Therapy

**FIGURE 5:**
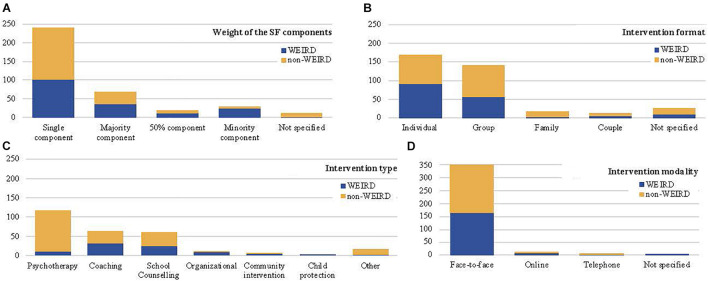
Comparison of SFBT features between WEIRD and non-WEIRD countries. **(A)** Weight of the SF components. **(B)** Intervention format. **(C)** Intervention type. **(D)** Intervention modality.

In the majority (84.38%) of the retrieved studies, the SFBT was either the exclusive component of the tested intervention (65.48%; 42.26% WEIRD and 57.74% non-WEIRD) or the main component (18.90%; 53.62% WEIRD and 46.38 non-WEIRD) (see Figure 5A). Only in a small proportion of studies the solution-focused component was one of two elements of the intervention (4.66%; 64.71% WEIRD and 35.29% non-WEIRD), or a minority component (8.2%; 86.21% WEIRD and 13.79% non-WEIRD). The differences between WEIRD and non-WEIRD countries in the overall distribution of the intervention content were statistically significant (*X*^2^ = 19.68; *p* = 0.000). *Z* analyses reveal that there were significantly more WEIRD than non-WEIRD publication on studies where the solution focused approach was a minority component (*Z* = 3.2; *p* < 0.05).

As far as the intervention type is concerned, a majority of the SFBT studied were classified as psychotherapy (56.44%), followed by coaching (16.99%) and school counseling (16.44%) (see Figure 5C). Less frequent were publications on SFBT with organizations (3.29%), communities (1.64%) and in child protection (0.82%); the contribution of non-WEIRD countries to the SFBT literature in these fields was almost inexistent (*n* = 6). The distribution of SFI type by WEIRD/non-WEIRD countries was not significantly different (*X*^2^ = 9.36; *p* = 0.154).

The intervention format of the SFBT interventions (see Figure 5B) was individual in 46.30% of the extracted studies (53.85% WEIRD and 46.15% non-WEIRD countries). Group interventions were the second most used interventions, with a 38.90% of the published papers (40.14% WEIRD and 59.86% non-WEIRD countries). Family SFBT was less frequent, 4.38% (12.50% WEIRD and 87.50% non-WEIRD), and couple SFBT was analyzed in 3.56% of the publications (38.46% WEIRD and 61.54% non-WEIRD). In 25 of the 365 SFBT research studies (4.38%) it was not possible to ascertain the intervention format. The differences between WEIRD and non-WEIRD countries in the distribution of SFI format were statistically significant (*X*^2^ = 16.63; *p* = 0.002). Z analyses reveal that there were significantly more non-WEIRD than WEIRD publication on group interventions (*Z* = 1.1; *p* < 0.05) and more WEIRD than non-WEIRD publications on family interventions (*Z* = 4.3; *p* < 0.05).

Regarding the intervention modality, an overwhelming majority of the SFBT took place face-to-face (95.89%), both in WEIRD (46.86%) and in non-WEIRD (53.14%) countries. Online (3.01%) and telephone (0.55%) interventions were rare (see Figure 5D).

### Design of the Retrieved Studies

As far as the scientific design of the extracted studies is concerned, 169 SFI outcome studies (46.30%) were randomized trials (36.69% WEIRD and 63.31% non-WEIRD). Quasi-experimental studies with non-randomized trials of two groups account for 26.85% of the publications (42.86% WEIRD and 57.14% non-WEIRD) and naturalistic, quasi-experimental single-group pre/post treatment studies for 13.42% (75.51% WEIRD and 24.49% non-WEIRD). The least frequent designs were single-case studies, with 6.85% of the publications (68% WEIRD and 32% non-WEIRD), and qualitative methodology studies, with 6.30% (73.91% WEIRD and 26.09% non-WEIRD). The differences between WEIRD and non-WEIRD countries were statistically significant (*X*^2^ = 34.75; *p* = 0.000). Z analyses reveal that there were significantly more RCTs in non-WEIRD than WEIRD countries (*Z* = 4.6; *p* < 0.05) whereas WEIRD studies were more often naturalistic (*Z* = 5.06; *p* < 0.05) or qualitative (*Z* = 3.4; *p* < 0.05) than non-WEIRD ones.

**FIGURE 6:**
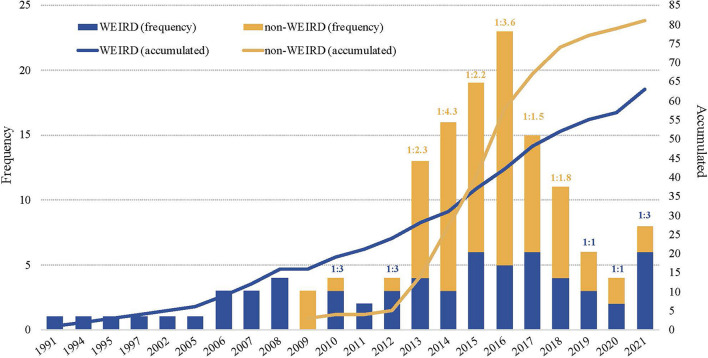
Temporal distribution of publications, accumulated frequency and WEIRD/non-WEIRD publication ratio in RCTs about SFBT.

Analyzing only RCTs with 30 or more experimental subjects (*n* = 144), the differences in the progression of studies in WEIRD vs. non-WEIRD (see Figure 6) became more accentuated than for the general data (see Figure 3). RCT on SFBT started to be published in WEIRD countries in 1991, while the first non-WEIRD RCTs appeared only in 2009. However, only 6 year later, in 2015, the SFBT studied with RCT in non-WEIRD countries had surpassed the level of RCTs in WEIRD countries, a trend that continues until 2021.

## Discussion

The first purpose of this study was to examine the development over time of the outcome research on SFBT. A second purpose was to determine if SFBT, which comes from a less theory-driven and more “hands-on” tradition than mainstream PPI, is WEIRD-centric from a scientometric point of view. Finally, we wanted to provide a bibliometric overview of authors, countries, and journals, and to give a broad description of the field in terms of the type of samples studied, the features of the interventions and the type of scientific designs employed to test them.

### General Bibliometric Data

In relation to our first purpose, our findings reveal an incremental growth in the number of outcome studies on SFBT over the last 30 years, showing an increasing interest in research on the effects of SFBT. We extracted 365 outcome research papers on SFBT published between 1991 and 2021, of which 169 were RCTs. This number comes close to the number of RCTs on PPIs than Hendriks et al. (2018) analyzed. In other words, although the solution-focused approach is less popular than mainstream PP, at the level of interventions it has generated a similar body of research. On the less bright side, only a minority of the SFBT outcome studies have been published in high impact journals, and in fact the two journals that have published the largest number of SFBT outcome papers are not even included in databases with high visibility. Another weakness of this body of research on SFI is that no strong networks of researchers seem to be operating in the field: our findings show that there are not many teams researching regularly on SFBT and that most authors are occasional authors, who have only published one research paper on SFI.

### Is Solution Focused Brief Therapy a WEIRD Approach?

Our results provide a clear answer to the second research question. In spite of the North American origin of SFBT, SFBT should not be considered a WEIRD approach: the outcome research on SFBT started later in non-WEIRD countries than in WEIRD countries, but since 2013 the yearly non-WEIRD production is doubling the WEIRD one. Therefore, non-WEIRD countries have already surpassed WEIRD countries in the accumulated number of research papers on the effects of SFBT, both for outcome research in general and for RCTs. Therefore, in bibliometric terms, SFBT is not a WEIRD practice and can be considered a global approach to intervention. This stands in contrast to the predominantly WEIRD nature of the research on PP (Hendriks et al., 2018; Kim et al., 2018). This difference between PP and SFBT in the globalization of outcome research can be seen as a reflection of the conceptual and practical differences between PP and SFBT that we highlighted in the introduction.

In our view, the expansion of outcome research on SFBT in non-WEIRD countries can be attributed to the atheoretical stance of the solution-focused approach, which allows using it in a diversity of cultural environments without the need of previous cultural adaptations. From this perspective, the fact that the solution-focused approach is basically procedural and content-free would make it suitable to address a variety of contents in a diversity of contexts. In fact, we only found two papers that performed an explicit cultural adaptation of SFBT (González et al., 2016; Stith et al., 2020). Furthermore, the finding that in most studies the solution-focused element was the only component of the tested SFI intervention confirms that the globalization of research on SFBT has not required *ad hoc* adaptations of the solution-focused procedures. This is different in the traditional PPIs, which are often adapted to specific populations (Hendriks et al., 2018).

In contrast to the globalization of research, it is noteworthy that while most WEIRD SFBT studies are published in English, only one-third of the outcome research on SFBT from non-WEIRD countries was published in that language. This relates to the scarcity of high-impact non-WEIRD publications on SFBT. It remains to be established if this is due to low quality of the published non-WEIRD research (that might be causing its rejection in more visible journals), to problems in the access of this research to English language journals, or to a lack of interest on the part of non-WEIRD authors to publish in these journals. In any case, the uneven distribution of WEIRD/non-WEIRD publication in language terms makes non-WEIRD research less accessible.

### Samples of the Extracted Studies

As far as other features of the tested SFBT interventions are concerned, there is a balanced distribution of the SFBT outcome research on clinical and non-clinical samples, with an almost identical number of studies on both. This confirms that the solution-focused approach has expanded well beyond the family therapy context in which it developed and is being applied in many other fields. In this respect, it would make sense to use the term “Solution-focused Interventions” or “Solution-focused Practice” (Sundman et al., 2019) instead of the somewhat narrower term “Solution-focused Brief *Therapy*.” No WEIRD/non-WEIRD differences were found in the distribution of SFI studies on clinical and non-clinical studies, suggesting that the expansion to non-clinical samples has happened globally.

College student samples constitute only 6.9% of the subjects that received the SFBT, which suggests that most of the outcome studies on SFBT have been done in the “real world,” outside university campuses. Given that American Psychology in general and PP in particular are sometimes critiqued as being based on skewed North American college samples (Arnett, 2008; Christopher and Hickinbottom, 2008; Frawley, 2015), our findings on SFBT provide a different picture.

### Features of the Interventions

Although non-clinical samples are well represented in the SFBT outcome research, more than half of the extracted SFBT papers were categorized as psychotherapy studies, followed in number by the studies on coaching and on school counseling. There are only few SFBT studies in the fields of child protection, organization development and community intervention, in spite of the fact that many authors and practitioners have convincingly presented the case for the application of solution-focused principles in these contexts (for instance, Sundman, 1997; Berg and Kelly, 2000; McKergow, 2012). In our view, the scarcity of the research on SFBT in child protection, organizations and communities does not necessarily mean that the solution focused approach is less useful in these contexts but could be explained by the difficulties to carry out effectiveness research in these fields.

As far as the format of interventions is concerned, the largest minority of the tested SFBT interventions were individual. “Group” was the second most frequent intervention format and was significantly more frequent in non-WEIRD than in WEIRD publications. Intriguingly, in spite of the fact that SFBT developed in a family therapy context (de Shazer et al., 1986), there were only a few publications on family SFBT interventions, more in WEIRD than in non-WEIRD countries. Although it has been argued that the solution-focused approach lends itself well to online interventions (Pakrosnis and Cepukiene, 2015), the overwhelming majority of the SFBT studied in the extracted papers were carried out face-to-face.

### Design of the Extracted Studies

Randomized controlled trials account for almost half of all outcome research papers on SFBT. We would like to highlight that the rhythm of publication of RCTs on SFBT in non-WEIRD countries has increased sharply over the last decade, so that non-WEIRD RCT publications currently outnumber WEIRD published RCTs. However, sample sizes are larger in WEIRD countries than in non-WEIRD ones, especially in research on clinical populations, with WEIRD samples almost 50% larger than non-WEIRD samples. This points to a possible weakness of non-WEIRD studies that may be making access to high impact journals more difficult.

### Limitations and Future Research

In our study the data extraction from the nine most relevant databases was complemented by a manual search in the SFBTA list and the 365 extracted publications were categorized according to a variety of dimensions. Alongside these strengths, there are also some weaknesses of our study. We have used a global categorization of WEIRD vs. non-WEIRD countries, ignoring possible regional differences within countries. It is also debatable to what extent the five dimensions encompassed by the WEIRD acronym should be given equal weight in the categorization as WEIRD or non-WEIRD. Given that our study covers a wide timespan of 30 years, with frequent political changes in some countries, we used an average of the Democracy Index. Therefore, our classifications of certain countries as WEIRD or non-WEIRD may not fit entirely with their current consideration as “full” vs. “flawed” democracies. In any case, the possible recategorization of some of these countries would not alter the overall results. We had no access to non-“western” databases, and a number of publications in Chinese and in Parsi, among others, could not be translated; therefore, non-WEIRD publications on SFBT may be actually under-represented.

Future bibliometric studies could attempt to include non-“western” data bases. Some possibly interesting variables, like the gender of the study population, could also be included. Another line for future bibliometric research would be to analyze citation trends for the retrieved articles.

Looking into the future of the outcome research on SFBT, our data suggest that it would be worthwhile to conduct more research on SFBT in child protection, community interventions and organizational interventions, both in WEIRD and non-WEIRD countries. Secondly, we look forward to seeing more outcome research on SFBT published in journals that are more visible for researchers and practitioners, especially for non-WEIRD publications. To that end, the reasons for the relative scarcity of non-WEIRD high-impact publications needs to be better understood, but in any case, an increase in the sample sizes of the SFBT outcome research might be helpful, especially for RCTs. Thirdly, higher team stability and longer research projects would contribute to a larger number of authors publishing more than only one paper on the outcomes of SFBT.

## Conclusion

Research on the effect of SFBT has been growing consistently over the last three decades. Almost half of this production are RCTs.

There are some important differences between WEIRD and non-WEIRD publications on SFBT in terms of the language of the publications and their visibility. Sample sizes are almost two times larger in WEIRD publications than in non-WEIRD publications, except for studies on school students; considering only RCT, the sample sizes of WEIRD publications triple those of non-WEIRD publications. SFBT with groups are more frequent in non-WEIRD than in WEIRD publications.

Although outcome studies on SFBT started in WEIRD countries, nowadays non-WEIRD publications on SFBT have quantitatively surpassed WEIRD research. Therefore, our findings support the statement that SFBT is not a WEIRD, but a global practice. Our results also confirm the wide applicability of the solution-focused approach in different fields. The number of SFBT papers on clinical and non-clinical samples is similar, and SFBT have been researched not only in the form of psychotherapy, but also as coaching and school interventions, with fewer SFBT outcome studies in organizations, child protection and communities.

## Data Availability Statement

The original contributions presented in the study are included in the article/supplementary material, further inquiries can be directed to the corresponding author/s.

## Author Contributions

MB, M-CN, BM-d-R, and ÁS-P: conceptualization, investigation, data curation, writing—original draft preparation, and writing—review and editing. M-CN and BM-d-R: methodology and formal analysis. BM-d-R and ÁS-P: visualization. All authors have read and agreed to the published version of the manuscript.

## Conflict of Interest

The authors declare that the research was conducted in the absence of any commercial or financial relationships that could be construed as a potential conflict of interest.

## Publisher’s Note

All claims expressed in this article are solely those of the authors and do not necessarily represent those of their affiliated organizations, or those of the publisher, the editors and the reviewers. Any product that may be evaluated in this article, or claim that may be made by its manufacturer, is not guaranteed or endorsed by the publisher.

## Footnotes

1
https://www.sfbta.org/current-research
